# SIRT1-Dependent Neuroprotection by Resveratrol in TOCP-Induced Spinal Cord Injury: Modulation of ER Stress and Autophagic Flux

**DOI:** 10.3390/toxics12110810

**Published:** 2024-11-11

**Authors:** Xiangsheng Tian, Yiquan Ou, Shengyuan Shi, Qiuhua Zhou, Sihong Long, Yao Xiang, Weichao Zhao, Dingxin Long

**Affiliations:** 1 Hunan Province Key Laboratory of Typical Environmental Pollution and Health Hazards, School of Public Health, University of South China, Hengyang 421001, China; txt190867082@gmail.com (X.T.); yiquanou@gmail.com (Y.O.); ssysyuans@gmail.com (S.S.); zhouqiuhua0224@163.com (Q.Z.); longsihong2000@163.com (S.L.); xbrainnet@163.com (Y.X.); zwcnhdx2019@163.com (W.Z.); 2 Department of Nutrition, The First People’s Hospital of Chenzhou, Chenzhou 423000, China

**Keywords:** resveratrol, neurotoxicity, tri-o-cresyl phosphate, sirtuin1, endoplasmic reticulum stress, autophagy

## Abstract

This study explores the neuroprotective effects of resveratrol (Resv) against tri-o-cresyl phosphate (TOCP)-induced neurotoxicity in the spinal cord of adult hens. It is well documented that TOCP exposure causes significant neurodegeneration via mechanisms that involve endoplasmic reticulum (ER) stress and impaired autophagy. In this experiment, adult hens were assigned to one of four groups: Control, Resv, TOCP, and TOCP + Resv. The spinal cord tissues were examined through transmission electron microscopy, hematoxylin and eosin (HE) staining, Nissl staining, and Western blotting to evaluate key proteins associated with ER stress and autophagy. Additionally, RT-qPCR and immunofluorescence were employed to measure sirtuin1 (SIRT1) expression. The findings revealed that TOCP induced severe ultrastructural damage, including disrupted myelin sheaths, dilated ER, and extensive neurodegeneration, as confirmed by histological evaluations. The expression levels of GRP78, p-PERK, p-eIF2α, ATF4, CHOP, Beclin-1, P62, and LC3-II were also significantly elevated by TOCP. However, Resv treatment markedly attenuated these pathological changes by reducing ER stress, restoring autophagic flux, and upregulating SIRT1 expression, preserving spinal cord integrity. These results indicate that Resv can effectively counteract TOCP-induced neurotoxicity by modulating ER stress and autophagy, underscoring its potential as a therapeutic agent for neuroprotection.

## 1. Introduction

**Figure 1:**
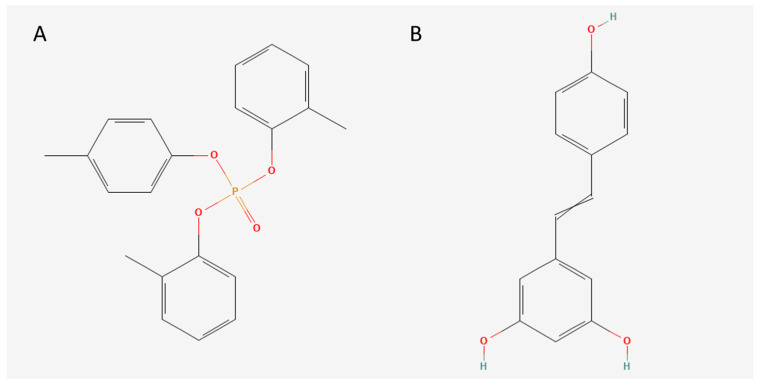
Chemical structure of TOCP and resveratrol. (**A**) TOCP (PubChem CID: 86003503). (**B**) Resveratrol (PubChem CID: 5056).

Tri-o-cresyl phosphate (TOCP) is an organophosphorus compound extensively employed in various industrial applications, such as a plasticizer, flame retardant, and lubricant additive. Additionally, TOCP is utilized as a solvent and is a component of jet engine oil [1]. Despite its industrial significance, TOCP has attracted considerable attention due to its potent neurotoxic effects. The chemical structure of TOCP, with its central phosphate group bonded to cresyl rings, is critical for its interaction with neuronal receptors and the development of neurotoxic effects (Figure 1A), particularly its role in inducing organophosphate-induced delayed neuropathy (OPIDN) [2]. Distal axonal degeneration of the central and peripheral nervous systems is a hallmark of this delayed neurotoxicity, resulting in serious neurological deficits, including ataxia and paralysis [3]. The pathophysiological mechanisms underlying TOCP-induced neurotoxicity are intricate, as it is involved in endoplasmic reticulum stress and various other cellular stress responses [4] and dysregulated autophagy [5], both of which are critical for maintaining neuronal homeostasis and function [6,7,8]. Clarifying the underlying molecular mechanics of these processes is crucial for developing therapeutic strategies aimed at mitigating TOCP-induced neurotoxicity.

The endoplasmic reticulum (ER) is critical for proper protein folding and processing. When its capacity is overwhelmed, misfolded or unfolded proteins accumulate in the ER lumen, triggering the unfolded protein response (UPR) [9,10,11]. By decreasing protein synthesis, increasing the ability to fold proteins, and encouraging the breakdown of misfolded proteins, the UPR aims to return the ER to its normal state. On the other hand, apoptosis may result from persistent or severe ER stress, especially if the UPR’s adaptive mechanisms are inadequate [8]. PERK is one of the primary signaling pathways of the UPR [12], and its activation initiates the PERK–eIF2α–ATF4–CHOP axis, which is central to converting ER stress into apoptotic signals [13]. PERK phosphorylates eIF2α, reducing overall protein synthesis while selectively increasing ATF4 translation. ATF4 upregulates genes that help restore ER homeostasis but also induces CHOP, which is a pro-apoptotic protein. This pathway is particularly relevant in the context of TOCP-induced neurotoxicity, where sustained ER stress can activate apoptotic pathways, leading to neuronal damage and degeneration.

Autophagy is a cellular degradation process essential for maintaining homeostasis by removing damaged organelles and misfolded proteins. Normally, cellular stress causes autophagy to be upregulated, including ER stress, as a protective mechanism [14]. It alleviates the burden of misfolded proteins by targeting them for degradation within lysosomes [15]. However, in the context of TOCP-induced neurotoxicity, the relationship between ER stress and autophagy becomes more complex. Chronic or excessive ER stress can impair autophagic flux, leading to the accumulation of autophagic substrates and exacerbating cellular dysfunction [16]. This impaired autophagic response contributes to the neurodegenerative processes observed in TOCP-induced toxicity. Research has demonstrated that TOCP can trigger autophagy in various cell types, including neurons [17,18,19]. Moreover, Long et al. has revealed the presence of autophagosomes containing degraded organelles in TOCP-treated cells, indicating that TOCP induces autophagy at the ultrastructural level [5]. However, while autophagy initially acts as a protective response, its prolonged activation under sustained ER stress conditions, as induced by TOCP, can lead to detrimental outcomes, contributing to neuronal cell death.

Resveratrol (Resv) is a polyphenol compound occurring in various plants, including knotweed and berries. Chemically, Resv exists as a stilbene derivative with two phenol rings connected by an ethylene bridge (Figure 1B). The chemical structure of Resv plays a crucial role in its biological activities, particularly its ability to scavenge reactive oxygen species (ROS) and modulate various signaling pathways involved in cellular stress responses [20]. Resv has gained recognition as a prospective neuroprotective agent, largely because of its capacity to activate Sirtuin1 (SIRT1), an NAD^+^-dependent deacetylase [21]. SIRT1 is a major regulator of cellular stress responses and is crucial in controlling the UPR and several key proteins involved in autophagy, enhancing the cell’s ability to cope with ER stress [22,23]. Specifically, SIRT1 deacetylates and activates several transcription factors and co-regulators, including ATF4 and PGC-1α [24], which are crucial in the cellular stress response. By promoting the deacetylation of these factors, SIRT1 enhances the adaptive capacity of the UPR, reducing the likelihood of ER stress-induced apoptosis. Furthermore, SIRT1 is pivotal in regulating autophagy by promoting autophagic flux through the deacetylation of key autophagy-related proteins [25]. This ability of SIRT1 in regulating both ER stress and autophagy positions it as a critical target for therapeutic intervention in conditions characterized by ER stress-induced neurotoxicity.

Adult hens are widely recognized as the most sensitive animal model for studying OPIDN, making them particularly suitable for investigating the neurotoxic effects of TOCP. The susceptibility of hens to OPIDN is well documented with clinical manifestations closely mirroring those observed in humans, including ataxia, paralysis, and distal axonal degeneration [26,27]. This similarity in pathophysiological responses positions hens as an ideal model for studying the underlying mechanisms of TOCP-induced neurotoxicity and for evaluating potential therapeutic interventions.

Our research aims to explore the role of Resv in mitigating TOCP-induced spinal cord neurotoxicity in adult hens. We hypothesized that Resv alleviates TOCP-induced neurotoxicity by activating SIRT1 to inhibit ER stress and restore autophagy flux. To test this hypothesis, we utilized a combination of histological, molecular, and biochemical analyses to evaluate the impact of Resv on ER stress markers, autophagy-related proteins, and neurodegenerative outcomes in the spinal cord of TOCP-exposed hens. Our results offer important new information on Resv’s potential as a therapeutic drug to reduce or eliminate organophosphate-related neurotoxicity.

## 2. Materials and Methods

### 2.1. Animal Treatment

**Figure 2:**
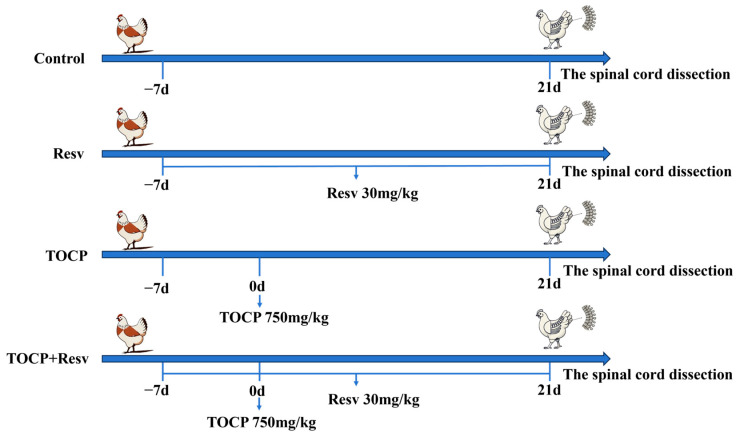
Schematic diagram of the experimental procedures.

Forty-eight healthy Xianghuang hens, aged 8 months, with a body weight ranging from 1.5 to 2.0 kg were selected for this study and purchased from Hengyang New Home Breeding Cooperatives. All animal operations were approved by the University of South China’s Laboratory Animal Ethics Committee (PN: USC2024-483) and treated humanely with as little pain as possible. The hens were given a week of adaptive feeding prior to the experiment in a typical laboratory setting where they had complete access to food and water. After acclimation, the hens were randomly divided into four groups, each comprising 12 hens: Control, Resv, TOCP, and TOCP + Resv (Figure 2).

- Control Group (*n* = 12): Hens in this group were administered empty capsules orally each day for the duration of the experiment.
- Resv Group (*n* = 12): Hens in this group received daily oral administration of capsules containing 30 mg/kg Resv (501-36-0, Aladdin, China).
- TOCP Group (*n* = 12): The hens in this particular group were given oral capsules containing 750 mg/kg of TOCP (78-30-8, Aladdin, China) as a single dosage.
- TOCP + Resv Group (*n* = 12): The hens in this group were given 30 mg/kg Resv orally every day in the form of capsules, starting 7 days prior to TOCP exposure. On the day of TOCP exposure, they received a single 750 mg/kg oral dose of TOCP, which was followed by continued daily Resv treatment for the remainder of the study. Capsules were specially formulated for ease of administration and to ensure accurate dosing. The capsules were composed of a biocompatible gelatin shell suitable for avian ingestion. Each capsule had a length of approximately 1.9 cm and a diameter of 0.66 cm, which was designed to be easily swallowed by the hens. Capsules were administered orally using a non-invasive gavage technique to ensure direct delivery to the stomach without causing stress or harm to the animals. The method of TOCP treatment is based on prior studies [28,29], while the dose of Resv was determined by an equivalent conversion of body surface area ratio [30] and supported by prior research [31,32].

Every day, the hens were observed for indications of neurotoxicity, and 21 days following their exposure to TOCP, the spinal cord was carefully extracted from the euthanized hens following humane euthanasia procedures in strict accordance with the Code of Ethics for laboratory animals.

### 2.2. Transmission Electron Microscopy (TEM)

The spinal cord tissues were preserved for 24 h at 4 °C using 2.5% glutaraldehyde in 0.1 M phosphate buffer (pH 7.4). After being washed in phosphate buffer, the fixed tissues were post-fixed in 1% osmium tetroxide for a duration of one to two hours at 4 °C. After being post-fixed, the tissues were cleaned in propylene oxide and dehydrated using a succession of ethanol grades (50, 70%, 90%, and 100%). After that, the tissues were polymerized at 60 °C and implanted in epoxy resin. Using an ultramicrotome, thin slices (70–90 nm) were cut, put on copper grids, and dyed with lead citrate and uranyl acetate. The ultrastructural changes in the spinal cord tissue were then assessed by transmission electron microscopy analysis of these sections.

### 2.3. Hematoxylin and Eosin (HE) Staining

Following dissection, the spinal cord tissues were preserved for 24 to 48 h at room temperature in 10% neutral-buffered formalin. Following fixation, the tissues were cleared in xylene, dehydrated using a sequence of increasing ethanol concentrations, and finally embedded in paraffin. Using a microtome, sections 5 µm thick were cut and then put onto glass slides for HE staining. After staining the sections, a light microscope was used to examine them for histological analysis. To enhance the rigor of our histopathological evaluations, we employed a semi-quantitative scoring system to assess neuronal damage. Damage was categorized based on the extent of nuclear pyknosis and vacuolization. Each slide was scored independently by three blinded observers on a scale from 0 (no damage) to 3 (severe damage), and the scores were averaged to obtain a final damage score for each sample.

### 2.4. Nissl Staining

After being preserved for 24 to 48 h in 10% neutral-buffered formalin, spinal cord tissues were dehydrated using a series of increasing ethanol concentrations and then embedded in paraffin. Five micrometer-thick sections were cut and put on glass slides. The sections were deparaffinized in xylene, rehydrated using a series of graded ethanol concentrations, and then washed in distilled water before being subjected to Nissl staining. After that, the slices were stained for five to ten minutes at room temperature using a 0.1% cresyl violet solution. Following staining, the sections underwent xylene clearing, 95% ethanol differentiation, 100% ethanol dehydration, and cover slip application of a synthetic mounting media. Neuronal density was assessed by counting Nissl-stained neurons with clearly visible Nissl bodies in randomly selected fields using light microscopy. We only counted neurons with a visible nucleus and Nissl granules, ensuring that fragmented or unclear neurons were not counted. The total number of neurons was manually counted, and the average number of neurons per field was calculated to estimate the neuronal density for each sample. The relative density of neurons was then compared between experimental groups. Neuron density is expressed as the number of neurons per unit area.

### 2.5. Immunofluorescence (IF)

The spinal cord tissues were cryopreserved in 30% sucrose in PBS until they sank, embedded in optimal cutting temperature (OCT) compound, and frozen at −80 °C after being fixed in 4% paraformaldehyde (PFA) for a whole night at 4 °C. Cryosections with a thickness of 10 to 15 µm were cut, permeabilized for 10 min in PBS containing 0.3% Triton X-100, and blocked for 1 h at room temperature in PBS containing 5% BSA. After washing and incubating with a fluorophore-conjugated secondary antibody for an hour at room temperature in the dark, sections were then treated with a primary SIRT1 antibody in 1% BSA/PBS for an overnight period at 4 °C. Sections were coverslipped, mounted with anti-fade media, and counterstained with DAPI following subsequent washing. To examine SIRT1 expression, the slides were examined under a fluorescence microscope.

### 2.6. Western Blot Analysis

Protease and phosphatase inhibitor-supplied RIPA lysis buffer was used to homogenize the tissues. To remove debris, the mixture was centrifuged at 15,000× *g* for 15 min at 4 °C. The bicinchoninic acid (BCA) assay was used to determine the total protein concentration after the resultant supernatants were collected. For the electrophoresis, 30 µg of total protein from each sample was loaded into each lane of the SDS-PAGE gel. After that, equal quantities of protein were separated using SDS-PAGE and put on PVDF membranes. The target proteins mentioned in Table 1 were the primary antibodies that were used to incubate the membranes for an overnight period at 4 °C after they had been blocked for two hours at room temperature with 5% skim milk. The membranes were washed with Tween-20 (TBS-T) and then treated for one hour at room temperature with secondary antibodies. An enhanced chemiluminescence (ECL) detection device was used to find immunoreactive bands, and ImageJ software was used to quantify them with GAPDH serving as the reference for normalizing protein expression.

### 2.7. Quantitative Real-Time PCR (RT-qPCR)

Using the FastPure Complex Tissue/Cell Total RNA Isolation Kit (RC113-01, Vazyme, Nanjing, China), the total RNA was extracted from the tissues. A spectrophotometer (Denovix, Wilmington, DE, USA) was used to measure the RNA’s purity and concentration. For qPCR, HiScript II Q RT SuperMix (R223-01, Vazyme, Nanjing, China) was used to create cDNA. The 7500 RT-PCR system (Thermo Fisher, Waltham, MA, USA) was used to conduct RT-qPCR. The ΔΔCt technique was used to evaluate the levels of gene expression. Table 2 lists specific primers for analysis.

### 2.8. Statistical Analysis

GraphPad Prism was used to analyze the experimental data, and mean ± SEM was used to represent each data set. To examine the differences between the groups, one-way ANOVA was employed, and Tukey’s post hoc test was utilized for multivariate comparison with a significance level of *p* < 0.05.

## 3. Results

### 3.1. TOCP-Induced Neurotoxicity in Adult Hens

**Figure 3:**
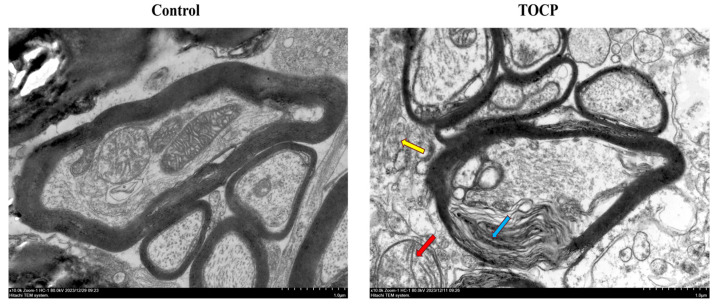
Effect of TOCP on ultrastructure of spinal cord in hens. Electron microscopy of spinal cord. Scale bar = 1.0 µm. The Control group represents a healthy nerve structure, the TOCP group indicates neurotoxicity, as evidenced by the disrupted myelin sheaths (blue arrow), the mitochondria exhibited disrupted cristae (red arrow) and dilated and disorganized ER (yellow arrow).

To assess the neurotoxic effects of TOCP, we examined ultrastructural changes in the spinal cord of adult hens using TEM (Figure 3). In the Control group, the axonal structures appeared intact with well-preserved myelin sheaths and clearly defined mitochondria. The ER exhibited a typical smooth and organized structure with no signs of stress or dilation. The ER membranes were closely associated with other organelles, maintaining their normal architecture and function, which is indicative of a healthy cellular environment. In contrast, the TOCP group showed a marked degeneration of the axonal structures. The myelin sheaths were irregular and displayed signs of decompaction, while the mitochondria exhibited disrupted cristae, which is indicative of early degenerative changes. Most notably, the ER in the TOCP-treated spinal cord displayed significant alterations, including dilation and disorganization of the ER membranes. These changes indicate ER stress, which is commonly associated with disrupted protein folding and increased cellular stress, contributing to the neurotoxic effects observed in the TOCP group. The pronounced ER disruption in the TOCP group underscores the impact of TOCP on intracellular organelle integrity, suggesting that a crucial part of the pathophysiology of TOCP-induced neurotoxicity may include ER stress.

### 3.2. Neuroprotective Effects of Resv on TOCP-Induced Neurotoxicity: Insights from HE Staining

**Figure 4:**
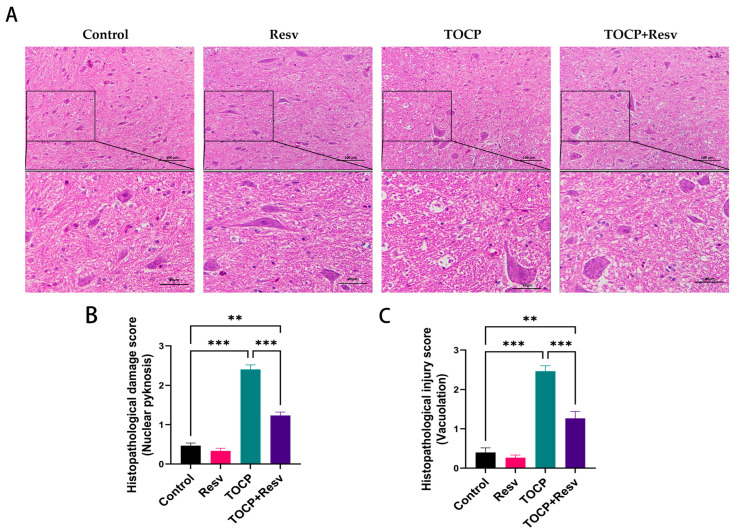
Resv can improve the spinal cord structural injury caused by TOCP. (**A**): HE staining of spinal cord (scale bar = 100 µm) and enlarged view of some areas (scale bar = 40 µm). (**B**): Histopathological score (nuclear pyknosis), *n* = 3. (**C**): Histopathological score (vacuolation), *n* = 3. Data are presented as the mean ± SEM. Statistical analyses were performed with one-way ANOVA. ** *p* < 0.01, *** *p* < 0.001.

The HE staining results (Figure 4A) revealed that the spinal cord in the Control group exhibited normal histological architecture with well-preserved neuronal structures and no signs of damage. Similarly, the Resv group showed a histological appearance comparable to the Control group, indicating that Resv treatment alone did not affect the structural integrity of the spinal cord. In contrast, the TOCP group demonstrated significant histopathological changes, including nuclear pyknosis, and noticeable vacuolization, which is indicative of severe neurodegeneration and tissue disruption caused by TOCP toxicity. However, in the TOCP + Resv group, the administration of Resv alongside TOCP resulted in a marked reduction in these pathological features. The neuronal structures in this group were better preserved with less vacuolization and cellular degeneration.

Histopathological damage score: nuclear pyknosis (Figure 4B) and vacuolation (Figure 4C) illustrate that the TOCP group showed significantly elevated scores compared to the Control and Resv groups, indicating substantial tissue damage as a result of TOCP exposure. The TOCP + Resv group had a marked reduction in both nuclear pyknosis and vacuolation scores compared to the TOCP group, demonstrating Resv’s protective effects against TOCP-induced damage. However, while Resv significantly reduces tissue injury, the TOCP + Resv group still exhibits higher damage and injury scores compared to the Control and Resv groups alone. This suggests that although Resveratrol ameliorates some of the histopathological damage caused by TOCP, it does not fully restore normal histology. According to the findings, Resv protects against TOCP-induced neurotoxicity by preserving spinal cord integrity and reducing the extent of histopathological damage.

### 3.3. Neuroprotective Effects of Resv on TOCP-Induced Neurotoxicity: Insights from Nissl Staining

**Figure 5:**
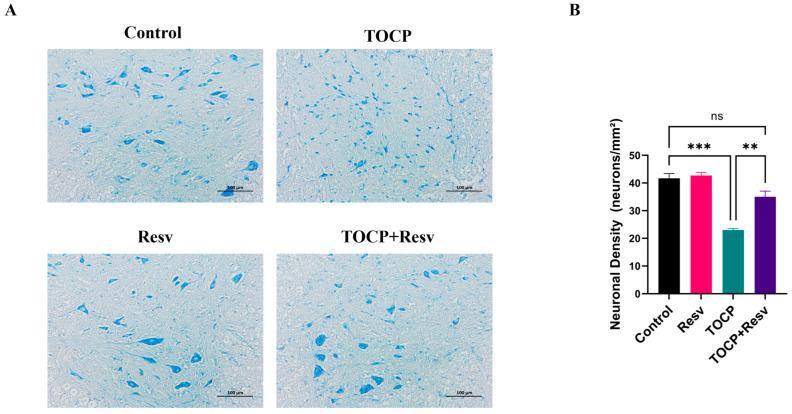
Resv has a protective effect on TOCP-induced neuronal injury. (**A**) Nissl staining of spinal cord. Scale bar = 100 µm. (**B**) Quantitative analysis of neuronal density (neurons/mm^2^), *n* = 3. Data are presented as the mean ± SEM. Statistical analyses were performed with one-way ANOVA. ** *p* < 0.01, *** *p* < 0.001, ns = not significant.

The Nissl staining results demonstrated significant differences in neuronal integrity among the various groups (Figure 5A). In the Control group, neurons exhibited a high density of Nissl bodies, with clear and well-defined staining, indicating healthy, intact neurons with robust protein synthesis. Similarly, the Resv group displayed strong Nissl staining comparable to the Control group, suggesting that Resv alone maintained neuronal integrity without inducing any observable neurotoxic effects. In contrast, the TOCP group showed a marked reduction in Nissl bodies with less intense staining and fewer discernible neurons, which is indicative of significant neurodegeneration and impaired protein synthesis caused by TOCP exposure. However, in the TOCP + Resv group, Resv treatment significantly ameliorated TOCP-induced neuronal damage as evidenced by the partial restoration of Nissl bodies and improved staining intensity. 

A quantitative analysis of neuronal density (neurons/mm^2^) further supported these findings (Figure 5B): while TOCP significantly reduced neuronal density compared to the Control, the TOCP + Resv group significantly mitigated this effect, resulting in a higher neuronal density compared to the TOCP group. The lack of a significant difference between the Control and Resv groups indicates that Resv does not inherently affect neuronal density, supporting its safety profile at the used concentration. This suggests that Resv possesses neuroprotective properties that are effective in the context of TOCP-induced neurotoxicity. These findings not only contribute to our understanding of Resv’s protective mechanisms but also highlight its potential therapeutic value in conditions involving organophosphate-induced neurotoxicity.

### 3.4. Resv Relieves TOCP-Induced ER Stress

**Figure 6:**
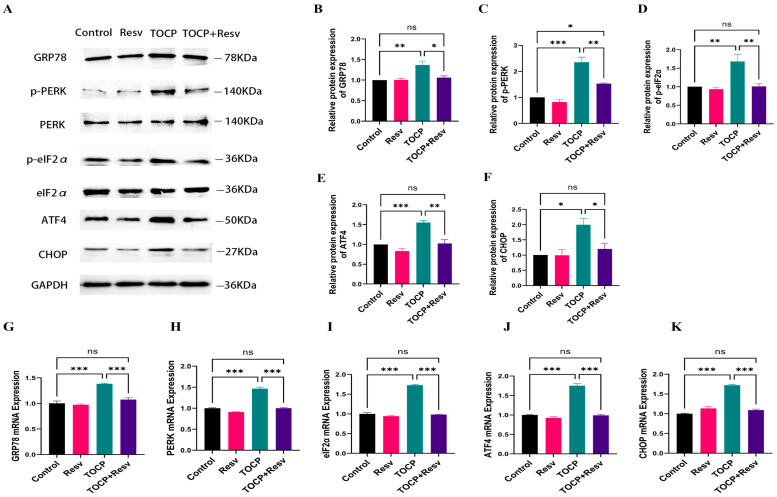
Resv can relieve ER stress induced by TOCP. (**A**) Western blotting analysis of ER stress-related proteins including GRP78, P-PERK, P-eIF2α, ATF4, and CHOP in spinal cord tissue. (**B**–**F**) Image J 1.50d was applied to analyze the gray scale of the protein bands of GRP78 (**B**), p-PERK (**C**), p-eIF2α (**D**), ATF4 (**E**) and CHOP (**F**) (*n* = 3). (**G**–**K**) The relative mRNA levels of GRP78 (**G**), PERK (**H**), eIF2α (**I**), ATF4 (**J**), and CHOP (**K**) in spinal cord tissue were detected using RT-qPCR (*n* = 4). Data are presented as the mean ± SEM. Statistical analyses were performed with one-way ANOVA. * *p* < 0.05, ** *p* < 0.01, *** *p* < 0.001, ns = not significant.

Given the substantial correlation between ER stress and TOCP-induced neurotoxicity, specifically through the PERK–eIF2α–ATF4 signaling axis, we looked at the impact of Resv on this pathway in the spinal cord. By using Western blot analysis (Figure 6A–F), it was possible to determine that TOCP exposure activated the ER stress pathway because the TOCP group showed a significant elevation of GRP78, p-PERK, p-eIF2α, ATF4, and CHOP protein levels in comparison to the Control group. However, in the TOCP + Resv group, Resv treatment significantly attenuated the TOCP-induced upregulation of these ER stress markers, although the levels did not fully return to those seen in the Control or Resv groups alone.

These results were corroborated by a study of mRNA expression (Figure 6G–K), which showed that the TOCP group had considerably higher mRNA levels of GRP78, PERK, eIF2α, ATF4, and CHOP than the Control group. This indicated increased transcriptional activation of the ER stress pathway. When comparing the TOCP + Resv group to the TOCP group, the mRNA expression of these markers was much lower, suggesting that Resv successfully downregulated ER stress at the transcriptional level. According to these findings, Resv may be able to reduce the cellular stress response and related neurotoxicity by influencing the protein and mRNA expression levels of important PERK–eIF2α–ATF4 pathway components, protecting against TOCP-induced ER stress.

### 3.5. Resv Modulates Abnormal Autophagy Flux Induced by TOCP

**Figure 7:**
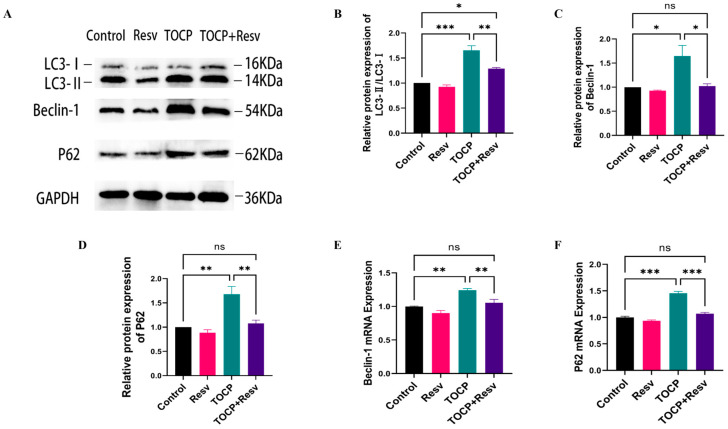
Resv modulates abnormal autophagy flux induced by TOCP. (**A**) Western blotting analysis of autophagy-related proteins including LC3-II, Beclin-1 and P62 in spinal cord tissue. (**B**–**D**) Image J was applied to analyze the gray scale of the protein bands of LC3-II (**B**), Beclin-1 (**C**), and P62 (**D**) (*n* = 3). (**E**,**F**) The relative mRNA levels of Beclin-1 (**E**), and P62 (**F**) in spinal cord tissue were detected using RT-qPCR (*n* = 4). Data are presented as the mean ± SEM. Statistical analyses were performed with one-way ANOVA. * *p* < 0.05, ** *p* < 0.01, *** *p* < 0.001, ns = not significant.

Our experiments have demonstrated that TOCP can induce ER stress. While ER stress can activate autophagy to degrade and recycle misfolded proteins and damaged organelles, persistent ER stress may impair autophagy flux, exacerbating ER stress and potentially creating a vicious cycle. This cycle, if left unchecked, can lead to reduced autophagy flux, cellular dysfunction, nerve damage, and other diseases. Therefore, we further investigated the effects of Resv and TOCP on autophagy. Comparing the TOCP group to the Control group, Western blot analysis revealed a significant elevation of the LC3-II, Beclin-1 and P62 protein levels (Figure 7A–D), indicating an accumulation of autophagy markers and suggesting disrupted autophagic flux due to TOCP exposure. In the TOCP + Resv group, Resv treatment significantly reduced the TOCP-induced upregulation of LC3-II, Beclin-1 and P62, indicating a partial restoration of autophagic balance. RT-qPCR analysis supported these protein-level findings (Figure 7E,F).

These results suggest that TOCP exposure disrupts autophagic processes, as evidenced by the accumulation of LC3-II, Beclin-1 and P62. However, Resv treatment helps mitigate these disruptions, likely by enhancing autophagic flux and reducing the stress-induced accumulation of autophagy markers. This indicates a potential protective role of Resv in maintaining autophagy homeostasis under neurotoxic conditions induced by TOCP.

### 3.6. Resv Activates SIRT1 and Mitigates TOCP-Induced Inhibition of SIRT1

**Figure 8:**
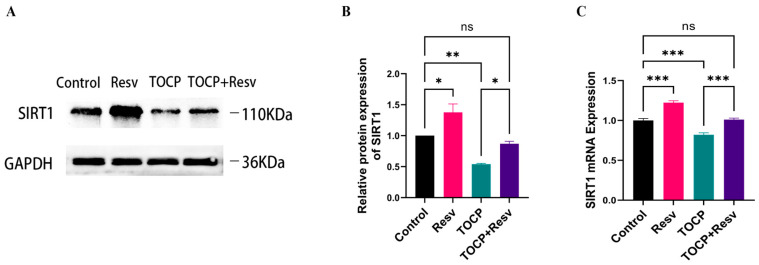
Resv can activate SIRT1 and reduce the inhibition of SIRT1 by TOCP. (**A**) Western blotting analysis of SIRT1 protein in spinal cord tissue. (**B**) Image J was applied to analyze the gray scale of the protein bands of SIRT1 (*n* = 3). (**C**) The relative mRNA levels of SIRT1 in spinal cord tissue were detected using RT-qPCR (*n* = 4). Data are presented as the mean ± SEM. Statistical analyses were performed with one-way ANOVA. * *p* < 0.05, ** *p* < 0.01, *** *p* < 0.001, ns = not significant.

We hypothesized that Resv alleviates TOCP-induced neurotoxicity by activating SIRT1. To verify this, we measured the expression levels of SIRT1 protein and mRNA in the Control, Resv, TOCP, and TOCP + Resv groups. Western blot analysis demonstrated a significant upregulation of SIRT1 protein expression in the Resv group. Conversely, the TOCP group exhibited a marked reduction in SIRT1 expression, indicating that TOCP exposure downregulated SIRT1 levels. Importantly, in the TOCP + Resv group, SIRT1 expression was notably recovered in comparison to the TOCP group (Figure 8A,B).

Consistent with these findings, the qPCR analysis demonstrated a similar trend in SIRT1 mRNA expression (Figure 8C). The Resv group’s SIRT1 mRNA levels were noticeably higher than those of the Control group, whereas the TOCP group exhibited a substantial decrease. In the TOCP + Resv group, SIRT1 mRNA levels were significantly elevated compared to the TOCP group, indicating that Resv treatment partially avoided the TOCP-induced downregulation of SIRT1.

**Figure 9:**
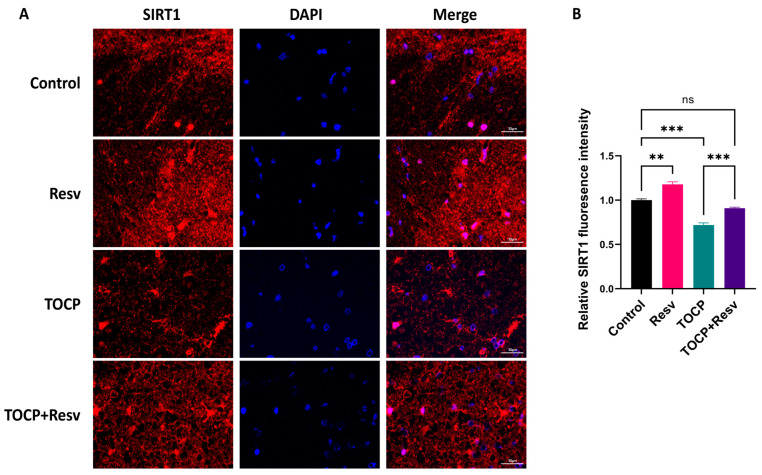
The expression and localization of SIRT1 in spinal cord were detected by immunofluorescence. (**A**) Immunofluorescence staining for SIRT1. Scale bar = 50 µm. (**B**) Quantitative analysis of SIRT1 fluorescence intensity (*n* = 3). Data are presented as the mean ± SEM. Statistical analyses were performed with one-way ANOVA. ** *p* < 0.01, *** *p* < 0.001, ns = not significant.

The SIRT1 immunofluorescence analysis revealed distinct variations in SIRT1 expression across the Control, Resv, TOCP, and TOCP + Resv groups (Figure 9A). In the Control group, SIRT1 fluorescence was uniformly distributed throughout the spinal cord tissue, indicating normal expression levels. The Resv group displayed a significant increase in SIRT1 fluorescence intensity, suggesting that Resv treatment upregulates SIRT1 expression. In contrast, the TOCP group showed a significant decrease in SIRT1 fluorescence intensity, indicating that TOCP exposure significantly downregulated SIRT1 expression, which could be associated with neurotoxicity. However, in the TOCP + Resv group, SIRT1 fluorescence intensity was significantly restored. The merged images with DAPI staining confirmed the localization of SIRT1 within the nuclei and cytoplasm of spinal cord cells. A quantitative analysis of SIRT1 fluorescence intensity further corroborated these observations, revealing statistically significant differences among the groups (Figure 9B).

These results imply that Resv has a preventive function against neurotoxicity caused by TOCP, likely mediated through the upregulation of SIRT1, which is a key protein involved in cellular stress responses and neuroprotection.

## 4. Discussion

The present study demonstrates that Resv exerts significant neuroprotective effects against TOCP-induced neurotoxicity in adult hens. This research provides novel insights into the mechanisms by which Resv mitigates the harmful effects of TOCP, particularly through its modulation of ER stress, autophagic flux, and SIRT1 signaling. These results align with and contribute to the expanding body of literature investigating the interplay of these pathways in neuroprotection [26,33,34].

TOCP-induced neurotoxicity is characterized by significant ultrastructural changes in the spinal cord, including myelin sheath degeneration, mitochondrial disruption, and pronounced ER dilation. These findings are consistent with previous studies [34,35] that have highlighted the neurotoxic potential of organophosphates like TOCP. For instance, Long et al. [5] found that TOCP induced autophagy in SH-SY5Y human neuroblastoma cells, which was closely associated with cytotoxicity and neural degeneration. Similarly, our study observed that TOCP exposure led to severe structural damage in the spinal cord, confirming the neurodegenerative impact of TOCP, as previously reported by Emerick et al. in their study on TOCP’s biochemical, histopathological, and clinical effects in hens [35].

The increased levels of GRP78, p-PERK, p-eIF2α, ATF4, and CHOP seen in the TOCP group indicate that the ER stress response is essential in mediating TOCP-induced neurotoxicity. These markers reflect a strong activation of the PERK pathway, which is a well-established mechanism that converts ER stress into apoptotic signals [36]. The findings from our study are consistent with those of Wan et al., who also reported that upregulation of the PERK pathway leads to lung injury and macrophage apoptosis [37]. Additionally, the study by Li et al. demonstrated a similar upregulation of ER stress markers in TOCP-treated cells, further supporting our observations [38].

Resv’s ability to mitigate TOCP-induced ER stress was one of the key findings of this study. The significant reduction in ER stress markers in the TOCP + Resv group suggests that Resv effectively attenuates the ER stress response, potentially preventing progression to apoptosis. This protective effect of Resv is consistent with previous studies that have demonstrated its role in modulating ER stress pathways. For example, Lou et al. [39] reported that Resv alleviates ER stress-induced cardiomyocyte apoptosis by downregulating the PERK–eIF2α–ATF4–CHOP pathway. Similarly, Pourhanifeh et al. found that Resv’s activation of SIRT1 played a crucial role in reducing ER stress and protecting against neurodegeneration [40].

The mechanistic insights provided by our study enhance the understanding of how Resv interacts with ER stress pathways. While prior research has primarily focused on in vitro models, our in vivo findings in hens offer valuable evidence that Resv’s modulation of ER stress is a robust and translatable therapeutic strategy. This is particularly important given the complexity of in vivo systems and the potential variability in Resv’s efficacy across different biological contexts.

Maintaining cellular homeostasis involves autophagy, which may be both a protective process and a potential cause of cell death when dysregulated [41,42,43]. In the context of TOCP-induced neurotoxicity, our study found that TOCP exposure disrupted autophagic flux, as evidenced by the accumulation of autophagy markers Beclin-1 and P62. These findings align with those of Chen et al. [33], who reported that TOCP-induced autophagy was associated with neural cytotoxicity and the breakdown of essential cytoskeletal components in SH-SY5Y cells.

The ability of Resv to restore autophagic balance in the TOCP + Resv group is a significant finding, suggesting that Resv not only mitigates ER stress but also enhances autophagic flux. This dual regulatory role of Resv aligns with the findings of other studies [44], which demonstrated that Resv could modulate autophagy to protect against neurotoxicity in an Alzheimer’s disease model. Resv’s ability to modulate autophagic flux and ER stress likely involves the regulation of key autophagy-related proteins, such as ATG5, ATG7, and ATG12, which are critical for autophagosome formation and maturation [45]. Resv has been shown to activate these ATG proteins, facilitating autophagy and preventing the accumulation of damaged organelles and misfolded proteins. In addition to ATG proteins, Resv also enhances lysosomal function, which is essential for the degradation of autophagic cargo [46]. Studies have demonstrated that Resv upregulates lysosomal markers, such as LAMP2 and Cathepsin D, further supporting its role in promoting autophagic flux and maintaining cellular homeostasis under stress conditions.

Furthermore, Resv has been found to regulate key signaling pathways involved in autophagy, including the mTOR and AMPK pathways [47]. By inhibiting mTOR and activating AMPK, Resv can induce autophagy and reduce cellular stress, thus protecting neurons from TOCP-induced neurodegeneration. These mechanistic insights highlight the multifaceted role of Resv in restoring autophagy and reducing ER stress, providing a more comprehensive understanding of how it protects against TOCP-induced neurotoxicity.

In addition to the modulation of ER stress and autophagy, the role of oxidative stress in TOCP neurotoxicity and the antioxidant properties of Resv also warrant discussion. Oxidative stress, characterized by an imbalance between the production of ROS and the body’s ability to counteract their harmful effects, is known to contribute to neuronal damage [48]. Resv’s known antioxidant properties may offer additional neuroprotective benefits by neutralizing ROS and enhancing cellular antioxidant defense mechanisms, providing a multi-targeted protective effect [49,50].

Additionally, the study by Marqués et al. [51] demonstrated that Resv’s effects on autophagy were mediated through the activation of SIRT1, further supporting the results of our study. Our study found that TOCP exposure resulted in a marked downregulation of SIRT1, which correlated with the observed neurotoxic effects. However, Resv treatment significantly restored SIRT1 expression levels in the TOCP + Resv group both at the protein and mRNA levels. This implies that the activation of SIRT1 is a crucial pathway through which Resv mediates its neuroprotective effects.

The literature has a wealth of information on SIRT1’s role in neuroprotection. For instance, Singh et al. [52] found that SIRT1 activation by Resv protected against neurodegeneration in a model of Parkinsonian disease by modulating autophagic flux and reducing ER stress. Similarly, our findings indicate that Resv’s neuroprotective effects in TOCP-induced neurotoxicity are at least partly mediated by SIRT1 activation. Further evidence is provided by Ren et al., who demonstrated that SIRT1 activation could increase cellular resistance to ER stress-induced apoptosis [53].

While our study focused on SIRT1 due to its well-established role in modulating neuroprotective pathways, including ER stress and autophagy, we recognize that the sirtuin family consists of several isoforms (SIRT1–SIRT7) with distinct biological functions [50,54]. Each isoform may play a role in cellular responses to stress, and their involvement in Resv-induced neuroprotection remains to be fully elucidated. Future studies should explore the potential contributions of other sirtuin isoforms, such as SIRT2 and SIRT3, to provide a more comprehensive understanding of the sirtuin family’s involvement in mitigating TOCP-induced neurotoxicity.

Our study’s findings shed fresh light on the neuroprotective mechanisms Resv uses to lessen the neurotoxicity caused by TOCP. First, the in vivo model used in this research underscores the translational potential of Resv as a potential neuroprotective agent for organophosphate-induced neurodegenerative diseases. The neuroprotective properties of Resv have been widely studied in mammalian models, including rodents: Resv’s ability to modulate autophagy, reduce oxidative stress, and activate pathways such as SIRT1 has been documented across various mammalian models of neuro-degenerative diseases, such as Alzheimer’s and Parkinson’s diseases. However, the hen model remains a valuable tool in studying OPIDN, as it closely mimics certain aspects of human exposure to organophosphates, including delayed neuropathic effects. Given the sensitivity of the hen model to TOCP-induced neurodegeneration, our study provides complementary insights into how resveratrol can protect against organophosphate-induced neurotoxicity.

Second, the dual role of Resv in modulating both ER stress and autophagy provides a comprehensive understanding of how Resv protects against neurotoxicity. This multifaceted approach is particularly important given the complexity of neurodegenerative diseases, where multiple cellular pathways are often dysregulated. By simultaneously targeting ER stress and autophagic flux, Resv presents a strong neuroprotective strategy that may be effective against various neurotoxic challenges.

Finally, the upregulation of SIRT1 as a key mediator of Resv’s effects underscores the potential of SIRT1 as a therapeutic target. Given that SIRT1 modulates both ER stress and autophagy, therapies that enhance SIRT1 activity could offer broad-spectrum protection against neurotoxicity. This is especially important given the context of organophosphate exposure, where there are currently limited effective therapeutic options.

## 5. Conclusions

In conclusion, this study provides compelling evidence that Resv offers significant neuroprotective benefits against TOCP-induced neurotoxicity in adult hens. By modulating ER stress, enhancing autophagic flux, and activating SIRT1, Resv mitigates the cellular damage induced by TOCP, preserving neuronal integrity and function. These findings are consistent with and build upon existing literature, further supporting the capacity of Resv as a treatment for neurodegenerative diseases associated with organophosphate toxicity. Subsequent research endeavors need to concentrate on converting these discoveries into practical scenarios and examining the enduring effects of Resv therapy in various neurodegenerative models.

## Figures and Tables

**Table 1:** The information of antibodies used in the present experiment.

Antibody	Clonality	Species	Dilutions	Company
SIRT1	Polyclonal	Rabbit	1:2000	Proteintech Group, Rosemont, IL, USA (13161-1-AP)
GRP78	Polyclonal	Rabbit	1:3000	Proteintech Group, Rosemont, IL, USA (11587-1-AP)
p-PERK	Polyclonal	Rabbit	1:1000	Affinity, Jiangsu, China (DF7576)
PERK	Polyclonal	Rabbit	1:1000	Proteintech Group, Rosemont, IL, USA (24390-1-AP)
p-eIF2α	Polyclonal	Rabbit	1:500	Affinity, Jiangsu, China (AF3087)
eIF2α	Polyclonal	Rabbit	1:500	Affinity, Jiangsu, China (AF6087)
ATF4	Polyclonal	Rabbit	1:1000	Proteintech Group, Rosemont, IL, USA (10835-1-AP)
CHOP	Polyclonal	Rabbit	1:500	Proteintech Group, Rosemont, IL, USA (15204-1-AP)
LC3-II	Polyclonal	Rabbit	1:2000	Abcam, Eugene, OR, USA (ab48394)
Beclin-1	Polyclonal	Rabbit	1:1000	Proteintech Group, Rosemont, IL, USA (11306-1-AP)
P62	Polyclonal	Rabbit	1:1000	ImmunoWay, Plano, TX, USA (YT7058)
GAPDH	Polyclonal	Rabbit	1:10,000	Proteintech Group, Rosemont, IL, USA (10494-1-AP)

**Table 2:** The primer sequences used for real-time PCR assay.

Gene	Forward Primer (5′-3′)	Reward Primer (5′-3′)
SIRT1	GCGGCTCGTGTCACAGTCAG	TCCTCAAATGCAGCTTCCACTTCC
GRP78	ACGGTTCGTGTGTGACGA	TAGGTGGTACCGAGGTCGATG
PERK	GGGCGAGGATGTTGTCTTAGTTGG	GCCGAGCAGATGTACTTCACCTTC
eIF2α	CGGAGGTGGAAGATGTTGTGATGG	CAGCTCACTGAGAAGGATCATGCC
ATF4	TCTGCAACCATGGCGTTT	AGGCTCATCTTGGTCAGGTTT
CHOP	CAACGGAGAATGAGCGGAGC	GCTCTTCCTTCTGGATGCCTTC
Beclin-1	GCAGGAAGAAGCTCAGTATCAG	CGCATCTGGTTCTCCACACTT
P62	CCAGGAACACAGCGAGTCAAGC	GGGATTCAATCAAGCGAGGGTCTG
GAPDH	CAGAACATCATCCCAGCGTCCAC	CGGCAGGTCAGGTCAACAACAG

## Data Availability

The data presented in this study are available on request from the corresponding author.
